# Perianal Crohn Disease in a Patient With Congenital Afibrinogenemia: A Multidisciplinary Therapeutic Management Challenge

**DOI:** 10.7759/cureus.112527

**Published:** 2026-07-12

**Authors:** Ayoub Bouziane, Ouiam Elmqaddam, Hajar Koulali, Abdelkrim Zazour, Zahi Ismaili, Ghizlane Kharrasse

**Affiliations:** 1 Department of Gastroenterology and Hepatology, Mohammed VI University Hospital, Oujda, MAR; 2 Research Laboratory of Pathology and Digestive Oncology, Faculty of Medicine and Pharmacy of Oujda, Mohammed First University, Oujda, MAR

**Keywords:** anti-tnf therapy, congenital afibrinogenemia, crohn disease, fibrinogen replacement therapy, perianal crohn disease

## Abstract

Congenital afibrinogenemia is a rare autosomal recessive coagulation disorder characterized by absent or severely reduced circulating fibrinogen levels, leading to variable hemorrhagic manifestations. The coexistence of congenital afibrinogenemia and Crohn disease is exceptionally rare and represents a major diagnostic and therapeutic challenge, particularly in patients with active fistulizing perianal disease requiring repeated invasive procedures and immunosuppressive therapy.

A 20-year-old woman with congenital afibrinogenemia diagnosed during childhood presented with chronic bloody diarrhea and perianal symptoms. Her bleeding history included umbilical cord bleeding, post-vaccination bleeding, recurrent ecchymoses, and fibrinogen levels below 0.4 IU/L. Seven months before admission, she developed a perianal abscess requiring surgical drainage, followed by persistent mucous and bloody diarrhea. Imaging demonstrated inflammatory rectal thickening and an active horseshoe anal fistula. Because of the anticipated hemorrhagic risk associated with endoscopic biopsies, intravenous fibrinogen concentrate was administered before endoscopy. The diagnosis of colonic Crohn disease with active fistulizing perianal involvement was established based on clinical, radiological, endoscopic, and histopathological findings. Following multidisciplinary evaluation, treatment with infliximab and azathioprine was initiated. During follow-up, recurrent epistaxis was successfully controlled with fibrinogen replacement therapy, while gastrointestinal and perianal symptoms markedly improved without major hemorrhagic, thrombotic, or infectious complications.

This association of congenital afibrinogenemia with active fistulizing perianal Crohn disease highlights the complexity of balancing inflammatory control, procedural safety, and hemorrhagic risk. Individualized multidisciplinary management and appropriate peri-procedural hemostatic support are essential to optimize outcomes in this exceptionally rare clinical setting.

## Introduction

Congenital afibrinogenemia is a rare autosomal recessive bleeding disorder with an estimated prevalence of approximately one per 1,000,000 individuals worldwide [1]. It is characterized by the absence or extremely low levels of circulating fibrinogen, leading to impaired coagulation and variable hemorrhagic manifestations [2]. In parallel, Crohn disease is a chronic inflammatory bowel disease characterized by transmural intestinal inflammation and heterogeneous clinical manifestations [3]. Perianal disease represents one of its most severe phenotypes and includes abscesses, fistulas, strictures, and chronic suppuration. Patients with perianal Crohn disease experience significant impairment in quality of life and often present a more aggressive disease course requiring intensified therapy and repeated surgical or endoscopic procedures [4,5]. In patients with an underlying severe bleeding disorder, these invasive repeated procedures may substantially increase the complexity of clinical management. Thus, the coexistence of Crohn disease and congenital afibrinogenemia represents an exceptionally rare diagnostic and therapeutic challenge.

We report the case of a young woman with congenital afibrinogenemia diagnosed with colonic Crohn disease and active fistulizing perianal disease, successfully managed with peri-procedural fibrinogen replacement therapy and combination treatment.

## Case presentation

A 20-year-old woman with congenital afibrinogenemia diagnosed during childhood presented with chronic bloody diarrhea and perianal symptoms. The diagnosis of afibrinogenemia had been established after recurrent bleeding manifestations since birth, including umbilical cord bleeding, post-vaccination bleeding, and recurrent ecchymoses, associated with fibrinogen levels below 0.4 IU/L. She previously received intermittent fresh frozen plasma transfusions.

Seven months before admission, she developed severe proctalgia followed by a perianal abscess requiring surgical drainage in January 2025. She subsequently developed chronic mucous and bloody diarrhea evolving into alternating bloody loose stools and constipation. Family history revealed ulcerative colitis in a maternal relative.

On admission, vital signs were normal. Proctologic examination revealed a fistulous opening. Lab tests showed microcytic iron-deficiency anemia (hemoglobin 9.7 g/dL, mean corpuscular volume (MCV) 75 fL), and vitamin D deficiency, whereas inflammatory markers remained low (CRP 3 mg/L) with a normal platelet count of 354 × 10⁹/L. Prothrombin time (PT) and activated partial thromboplastin time (aPTT) could not be measured because the laboratory reported that the blood sample was non-coagulable.

CT enterography demonstrated circumferential inflammatory thickening of the lower rectum with partial regression of gluteal and perineal collections following previous surgical drainage (Figure 1). Pelvic MRI revealed an active horseshoe anal fistula extending from the 7 o’clock position to the left intergluteal fold (Figure 2).

**Figure 1:**
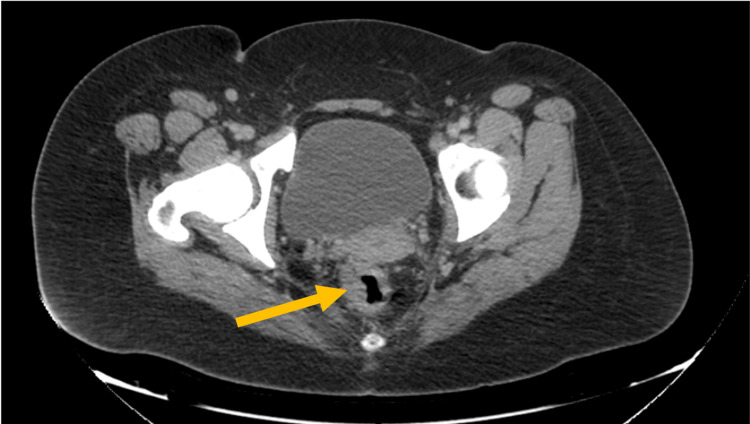
CT enterography demonstrating circumferential inflammatory thickening of the lower rectum with partial regression of gluteal and perineal collections following previous surgical drainage of a perianal abscess.

**Figure 2:**
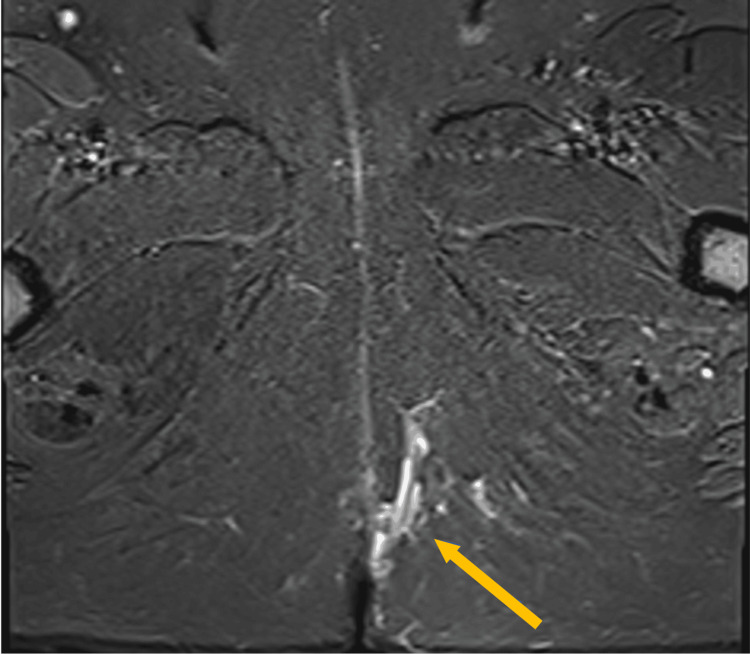
Coronal short tau inversion recovery (STIR) pelvic MRI showing inflammatory changes consistent with active perianal disease.

Because of the anticipated bleeding risk related to endoscopic biopsies, peri-procedural hemostatic management was performed in collaboration with the internal medicine team. A total of 6 g of intravenous fibrinogen concentrate was administered before endoscopy. Post-infusion laboratory testing showed a fibrinogen level of 2.0 g/L, PT of 79% (international normalized ratio (INR) 1.14), and a normalized aPTT (ratio 1.0), allowing safe endoscopic evaluation with mucosal biopsies. Colonoscopy showed erythematous congestive inflammation involving the rectum, right colon, and transverse colon, with diffuse loss of vascular pattern and no skip areas. The left colon, sigmoid colon, and terminal ileum appeared macroscopically normal. Upper gastrointestinal endoscopy was unremarkable. Histopathology demonstrated chronic inflammatory changes with architectural distortion, focal basal plasmacytosis, cryptitis, and crypt abscesses. No granulomas, dysplasia, malignancy, or infectious agents were identified. The differential diagnosis included ulcerative colitis because of the continuous colonic involvement and infectious colitis. However, the presence of an active complex perianal fistula, together with the overall clinical, radiological, endoscopic, and histopathological findings, favored the diagnosis of Crohn disease.

Following multidisciplinary evaluation, the diagnosis of colonic Crohn disease with active fistulizing perianal involvement was established. Treatment with infliximab 5 mg/kg and azathioprine 150 mg daily was initiated. During follow-up, the patient developed recurrent epistaxis successfully managed with fibrinogen replacement therapy. Nevertheless, she achieved sustained clinical remission with marked improvement of gastrointestinal and perianal symptoms.

## Discussion

Congenital afibrinogenemia is a rare autosomal recessive bleeding disorder characterized by the complete absence of fibrinogen, a key component of coagulation and clot formation. Bleeding manifestations usually begin early in life and may occur spontaneously or following trauma or invasive procedures. Umbilical cord bleeding is the most frequent neonatal presentation. Other manifestations include mucosal bleeding, ecchymoses, hemarthrosis, and musculoskeletal bleeding, whereas gastrointestinal bleeding remains uncommon. The diagnosis is established by undetectable fibrinogen activity associated with absent or extremely low fibrinogen antigen levels [6]. Current recommendations emphasize fibrinogen replacement therapy for both acute bleeding episodes and peri-procedural prophylaxis. Target fibrinogen levels generally exceed 1 g/L for minor bleeding and 1.5 g/L for major bleeding or invasive procedures. Administration of 50-70 mg/kg of fibrinogen concentrate usually increases plasma fibrinogen levels by approximately 1 g/L, with an estimated half-life of three to four days [1].

Afibrinogenemia may be associated with other diseases. Herein, we describe, to our knowledge, the first published case of afibrinogenemia associated with Crohn disease. This association is particularly challenging, as both conditions have specific management requirements and potential complications. Their coexistence may lead to overlapping complications, complex therapeutic decision-making, and possible drug-related effects or interactions. Gastrointestinal bleeding is uncommon in both Crohn disease and congenital afibrinogenemia [6]. However, in patients presenting with both conditions, the underlying coagulation disorder may accentuate intestinal bleeding and contribute to an atypical presentation, potentially delaying the diagnosis of inflammatory bowel disease. In our patient, diagnosis was further complicated by the hemorrhagic risk associated with endoscopic biopsies, which remain essential for confirming inflammatory bowel disease and assessing disease extent. Because endoscopic procedures with biopsies are considered high hemorrhagic risk procedures, prophylactic fibrinogen replacement therapy was administered before colonoscopy and upper gastrointestinal endoscopy [1]. Given the anticipated procedural bleeding risk and the absence of baseline fibrinogen activity before endoscopy, a target fibrinogen level of approximately 2 g/L was selected. Consequently, 6 g of intravenous fibrinogen concentrate was administered before the procedure. This strategy allowed safe endoscopic evaluation and mucosal biopsies without immediate hemorrhagic complications.

The hemorrhagic challenge becomes even more relevant in active fistulizing perianal Crohn disease, where repeated interventions such as abscess drainage, seton placement, fistula management, and repeated endoscopic procedures are frequently required. Consequently, this association requires close coordination between gastroenterologists, surgeons, radiologists, and hematologists. Management of perianal Crohn disease relies on a multidisciplinary approach combining surgical drainage and biologic therapy. Early anti-tumor necrosis factor (TNF) therapy, particularly infliximab, significantly improves fistula healing and long-term disease control [7]. Combination therapy with immunosuppressants may further improve sustained response by reducing immunogenicity [8]. In our patient, bleeding risk was mainly procedure-related, with no history of severe spontaneous hemorrhage requiring regular prophylactic replacement therapy. Therefore, non-invasive monitoring using validated clinical, biological, and imaging markers should be prioritized whenever possible. This delicate balance between inflammatory control, infectious risk, and hemorrhagic risk represents the major therapeutic challenge in such patients. The cost and limited availability of fibrinogen concentrate may represent an additional challenge, particularly in resource-limited settings. Therapeutic decisions must therefore balance disease control, procedural safety, treatment accessibility, and long-term hemorrhagic risk.

The decision to initiate infliximab and azathioprine was made after multidisciplinary discussion and careful evaluation of the benefit-risk balance. During follow-up, recurrent epistaxis was successfully managed with fibrinogen replacement therapy. To the best of our knowledge, no clinically relevant pharmacological interactions between fibrinogen replacement therapy and infliximab or azathioprine have been reported in the available literature. During the 9-month follow-up, the patient progressively improved under treatment, with resolution of diarrhea and marked improvement of perianal symptoms. Hemoglobin levels increased from 9.7 g/dL to 12.6 g/dL, while CRP remained low (1.7mg/L) and platelet count remained within the normal range (257 × 10⁹/L). No major hemorrhagic, thrombotic, or severe infectious complications were observed during follow-up. Importantly, no worsening of hemorrhagic manifestations occurred after anti-TNF initiation, suggesting that biologic therapy may be feasible in selected patients with congenital coagulation disorders under careful monitoring and appropriate hemostatic support.

To the best of our knowledge, no similar case involving active fistulizing perianal Crohn disease associated with congenital afibrinogenemia has previously been reported. Nevertheless, several reports have described inflammatory bowel disease associated with other inherited or acquired bleeding disorders, as summarized in Table 1 [9-11]. This comparison places the present case in the context of the available literature and highlights the uniqueness of this association. This report remains limited by its single-case design and the absence of specific recommendations for managing patients with concomitant Crohn disease and congenital afibrinogenemia. Additional reports and collaborative experience are needed to better define optimal diagnostic, hemostatic, and therapeutic strategies for this exceptionally rare association.

**Table 1:** Published cases of inflammatory bowel disease associated with rare inherited or acquired bleeding disorders and their relevance to the present case. FVIII: factor VIII; GI: gastrointestinal; IBD: inflammatory bowel disease; UC: ulcerative colitis; vWF: von Willebrand factor

Case report	IBD type	Bleeding disorder	Main presentation	Management	Key message
Pratap and Foord, 2026 [9]	Indeterminate colitis/UC-like colitis	Previously undiagnosed Congenital von Willebrand disease (vWF)	Chronic rectal bleeding, anemia, later excessive surgical bleeding	IBD therapy, surgery, then on-demand and prophylactic vWF replacement; rectal bleeding resolved after vWF replacement	Persistent or disproportionate GI bleeding in IBD should prompt evaluation for an associated bleeding disorder.
Di Sabatino et al., 2017 [10]	Crohn disease and ulcerative colitis	Acquired von Willebrand syndrome	Epistaxis, macrohematuria, muscle hematoma	vWF/FVIII-containing concentrates used for bleeding assessment and management	New bleeding symptoms in IBD should not automatically be attributed to intestinal inflammation; coagulation testing is important.
Soares et al., 2026 [11]	Crohn disease	Previously undiagnosed mild hemophilia A	Recurrent massive lower GI bleeding, hemodynamic instability, surgery	Blood transfusion, Fresh Frozen Plasma, surgery, biologic therapy; later prophylactic factor VIII before endoscopic/surgical procedures	Severe or recurrent bleeding in Crohn disease requires systematic evaluation for coagulation disorders and individualized peri-procedural replacement therapy.
Present case	Colonic Crohn disease with perianal involvement	Congenital afibrinogenemia	Chronic bloody diarrhea and perianal disease	Peri-procedural fibrinogen replacement before endoscopy, followed by infliximab and azathioprine	Combination therapy can be feasible with multidisciplinary management and targeted hemostatic support.

## Conclusions

This first reported case of congenital afibrinogenemia associated with Crohn disease highlights the complexity of managing two rare concomitant conditions, particularly in the setting of active fistulizing perianal disease requiring repeated invasive procedures and escalation to combination immunosuppressive therapy. In our patient, the diagnosis of Crohn disease was favored based on the overall clinical, radiological, endoscopic, histopathological, and multidisciplinary assessment. This case underscores the importance of individualized multidisciplinary management involving gastroenterologists, hematologists, surgeons, radiologists, and pathologists to achieve adequate inflammatory control while minimizing hemorrhagic and infectious complications and, whenever feasible, limiting invasive procedures. Given the absence of specific recommendations for this exceptionally rare clinical association, this report may contribute to improving awareness and guiding therapeutic decision-making in similar complex clinical situations.
